# Gradient boosting machine learning model to predict aflatoxins in Iowa corn

**DOI:** 10.3389/fmicb.2023.1248772

**Published:** 2023-09-01

**Authors:** Emily H. Branstad-Spates, Lina Castano-Duque, Gretchen A. Mosher, Charles R. Hurburgh, Phillip Owens, Edwin Winzeler, Kanniah Rajasekaran, Erin L. Bowers

**Affiliations:** ^1^Department of Agricultural and Biosystems Engineering, Iowa State University, Ames, IA, United States; ^2^USDA, Agriculture Research Service, Southern Regional Research Center, New Orleans, LA, United States; ^3^USDA, Agriculture Research Service, Dale Bumpers Small Farms Research Center, Booneville, AR, United States

**Keywords:** aflatoxin, gradient boosting, Iowa, corn, feed safety, prediction modeling

## Abstract

**Introduction:**

Aflatoxin (AFL), a secondary metabolite produced from filamentous fungi, contaminates corn, posing significant health and safety hazards for humans and livestock through toxigenic and carcinogenic effects. Corn is widely used as an essential commodity for food, feed, fuel, and export markets; therefore, AFL mitigation is necessary to ensure food and feed safety within the United States (US) and elsewhere in the world. In this case study, an Iowa-centric model was developed to predict AFL contamination using historical corn contamination, meteorological, satellite, and soil property data in the largest corn-producing state in the US.

**Methods:**

We evaluated the performance of AFL prediction with gradient boosting machine (GBM) learning and feature engineering in Iowa corn for two AFL risk thresholds for high contamination events: 20-ppb and 5-ppb. A 90%–10% training-to-testing ratio was utilized in 2010, 2011, 2012, and 2021 (*n* = 630), with independent validation using the year 2020 (*n* = 376).

**Results:**

The GBM model had an overall accuracy of 96.77% for AFL with a balanced accuracy of 50.00% for a 20-ppb risk threshold, whereas GBM had an overall accuracy of 90.32% with a balanced accuracy of 64.88% for a 5-ppb threshold. The GBM model had a low power to detect high AFL contamination events, resulting in a low sensitivity rate. Analyses for AFL showed satellite-acquired vegetative index during August significantly improved the prediction of corn contamination at the end of the growing season for both risk thresholds. Prediction of high AFL contamination levels was linked to aflatoxin risk indices (ARI) in May. However, ARI in July was an influential factor for the 5-ppb threshold but not for the 20-ppb threshold. Similarly, latitude was an influential factor for the 20-ppb threshold but not the 5-ppb threshold. Furthermore, soil-saturated hydraulic conductivity (Ksat) influenced both risk thresholds.

**Discussion:**

Developing these AFL prediction models is practical and implementable in commodity grain handling environments to achieve the goal of preventative rather than reactive mitigations. Finding predictors that influence AFL risk annually is an important cost-effective risk tool and, therefore, is a high priority to ensure hazard management and optimal grain utilization to maximize the utility of the nation’s corn crop.

## 1. Introduction

Aflatoxin (AFL), a type of mycotoxin, is produced in cereal grains, such as corn, as secondary metabolites from certain types of fungi on plants. Corn is susceptible to AFL toxigenic strains, which pose a significant health, economic, and safety risk to humans and livestock when they consume contaminated products (Munkvold et al., 2019). The economic impact of AFL has been estimated to be between $418 million to $1.66 billion for all stakeholders in the US agricultural industry and can infiltrate the supply chain in corn-based commodities (Wu, 2006; Mitchell et al., 2016). AFL is primarily produced from the fungal strains *Aspergillus flavus* and *A. parasiticus* via the polyketide pathway (Sweeney and Dobson, 1998). Contamination can happen during any stage, from in the field pre-harvest, during the growing season, harvest, and in post-harvest storage (Payne and Widstrom, 1992).

Extensive literature has been published on environmental conditions conducive to producing AFL in corn, with specific conditions that favor production and development (Diener et al., 1987; Payne and Widstrom, 1992; Cotty and Jaime-Garcia, 2007; Windham et al., 2009). Three specific factors are needed to create the right conditions for a pathogen to invade plants, create disease, and produce mycotoxins. This is known as the traditional balanced triangle between (1) the pathogen and pest, and (2) host, and (3) environmental conditions (Medina et al., 2017; Perrone et al., 2020). *A. flavus* and *parasiticus* have been documented to cause infection under drought conditions in dry, hot weather ranging from 29 to 35°C (Schindler et al., 1967; Payne, 1998). Additionally, AFL infection will likely develop when these high temperatures continue through the nighttime period without proper cooldown (Diener et al., 1987; CAST, 2003). Corn is susceptible to AFL infection through the ear silks, with stress conditions at pollination increasing the chance of plant disease (Marsh and Payne, 1984; Damianidis et al., 2018). Furthermore, soil is often the reservoir for *Aspergillus*, while insect vectors, direct contact, or dust can transmit spores (Winter and Pereg, 2019). The distribution and growth of AFL in soil depend on many factors, including geographical region, soil type, water retention rate, climatic conditions, crop rotation, and insect presence (Zhang et al., 2017; Winter and Pereg, 2019). Elevated soil temperatures have been positively correlated to AFL contamination and the degree to which insect activity impacts AFL content regionally; however, more literature is necessary regarding soil properties and how they influence fungal growth (Bilgrami and Choudhary, 1998; Payne, 1998). Temperature and rainfall conditions in the principal corn-growing states in the US are typically sufficient to slow the growth of *A. flavus* and *parasiticus,* avoiding significant AFL accumulation (Munkvold, 2014). However, in drought and high-temperature years, AFL contamination has been documented in Iowa (Lillehoj et al., 1976; Schmitt and Hurburgh, 1989; Mitchell et al., 2016). These AFL challenges exist in the Corn Belt region of the US; climate change patterns with temperature increases will likely increase the AFL concentration in corn in the US (Wu et al., 2011; Yu et al., 2022).

The Food Safety Modernization Act (FSMA) legislation warrants stakeholders to be preventative versus reactive with food and feed safety events, including mycotoxin outbreaks of AFL in corn (Grover et al., 2016). Therefore, AFL prediction and risk assessment systems that alert stakeholders of possible outbreaks are essential. Wu et al. (2011) stated, “Quantitative, site-specific risk assessments or predictive models for mycotoxin accumulation could contribute significantly to management efficiency in maize.” While many efforts have been undertaken to predict AFL by leveraging climate and weather data and interactions with crop developmental phases, the models are often based on generating new datasets, *in vitro* data, or conducted in other regions of the world (Johansson et al., 2006; Probst and Cotty, 2012; Leggieri et al., 2015; Battilani et al., 2016; Smith et al., 2016). These models are generally not applicable to the US corn growers, grain handlers, processors, and end-users due to differences in geographical location, management practices, weather, and predictions of mycotoxin contamination with decreased accuracy levels (de Schrijver et al., 2021; Castano-Duque et al., 2022). The European models established a general framework for a US-specific model with mycotoxin corn predictions (Battilani et al., 2013; Van der Fels-Klerx et al., 2019). Castano-Duque et al. (2022) developed the first US machine-learning models using feature engineering in combination with gradient boosting machine (GBM) learning and Bayesian networks to predict AFL contamination in Illinois-grown corn concerning weather and plant-related parameters such as vegetative index, aflatoxin risk index (ARI), and climate zones.

With Iowa being the top corn-producing state in the US, thoughtful, comprehensive, and strategic management solutions, such as predicting AFL contamination on an annual and localized basis created for grain processors and handlers, allow for appropriate decision-making in a preventative versus reactive manner (USDA-NASS, 2023a,b). The development of prediction models can enable early action to prevent or hinder mycotoxin development through integrated solutions that are controllable such as early harvest of at-risk grain, isolation of contaminated grain, application of fungicides, drying to lower storage moistures, and strategic marketing to more tolerant end users (Fumagalli et al., 2021). For grain elevators, handlers, and processors, prediction models enable proactive planning for handling, storing, and marketing grain with differing risk levels and facilitates strategic sampling and testing (Fumagalli et al., 2021). These machine-learning models can guide rapid decision-making and diversion necessary before the point of first receipt at the elevator to improve the overall safety and profitability of the US corn supply without compromising the profitability of individual grain businesses (Mitchell et al., 2016; Castano-Duque et al., 2022).

The main objective of this study aimed to evaluate the performance of AFL prediction with GBM models and feature engineering in Iowa corn with two risk thresholds: 20-ppb and 5-ppb. Historical climate data, soil property data, and historical Iowa AFL data collected in 2010, 2011, 2012, and 2021 were used in the GBM model. The combination of historical climate, weather, soil property data, and AFL contamination data in Iowa helped determine indicators of risk preharvest. AFL risk predictions from the Iowa-centric model provide a baseline for indicating disease in the corn crop, paving the way for further development of proactive actions and decisions that grain supply chain stakeholders can adopt for AFL mitigation control.

## 2. Materials and methods

### 2.1. Mycotoxin, weather, and soil property data

Historical AFL contamination data for corn was obtained from the Iowa Department of Agriculture and Land Stewardship (IDALS) for 99 counties from 2010, 2011, 2012, and 2021. County-level data was unavailable for 2013–2019, as it was reported on a different geographic scale (i.e., Crop Reporting District) in Iowa and was incompatible with county-level weather and crop developmental parameters. Data from 2020 were reserved for internal model validation collected from the same source. IDALS conducts annual statewide surveys of mycotoxin occurrence in Iowa corn. The current state sampling plan requires at least one corn sample and up to four samples collected annually from one elevator or processor in Iowa’s 99 counties during the harvest season.

In 2010, 2011, and 2021, two corn samples were collected from each of the 99 counties’ grain-handling facilities (1,360.78–4,535.92 g/sample). In 2012 and 2020, sampling was ramped up to four corn samples (1,360.78–4,535.92 g/sample) collected from each grain handling facility (grain elevators and cooperatives) in Iowa’s 99 counties. Samples were collected from the scale-house probe grain depositories, as it was additionally collected from incoming corn loads for grading purposes. The corn samples analyzed represent mixtures of the loads received on the day they were collected. Samples at IDALS were ground using a Romer Series II sub-sampling mill. The output sub-sample was mixed, and a test portion was selected and analyzed using AgraQuant ELISA Total Aflatoxin Assay (B_1_ + B_2_ + G_1_ + G_2_) (COKAQ1000 4–40 ppb) (Romer Laboratories, Union, MO, United States), according to manufacturer instructions. Mycotoxin quantification methods detect and report the sum of aflatoxins B_1_, B_2_, G_1_, and G_2_.

Historical monthly average temperature and precipitation data were obtained from the National Oceanic and Atmospheric Administration (NOAA),^1^ and the monthly vegetative index was obtained from GRO-Intelligence.^2^ The vegetative index was calculated from satellite data sensors that detect the intensity of NIR and visible red light reflected. These values are used to calculate the normalized difference vegetative index (NDVI), therefore, measuring plant greenness in Iowa. Forty-eight soil properties were used as predictors in the model obtained from digital soil mapping from USDA-NRCS soil survey data (Walkinshaw et al., 2022; USDA-NRCS, 2023; Supplementary Table S1). Historic meteorological data was linked to county-level AFL data using the county and year as common information. Six hundred thirty-nine points were obtained for AFL data for Iowa’s 99 counties. After linking the weather and AFL occurrence data, the data was reduced to 630 observations. Some data were eliminated due to insufficient historical average monthly weather data for two counties, Adams and Wright, from NOAA in 2012.

### 2.2. Features engineering and imputation for AFL dataset

Monthly precipitation and temperature data were averaged per county for all 4 years: 2010, 2011, 2012, and 2021. The average temperatures (T) and precipitation were obtained from NOAA in degrees Celsius (°C) and millimeters (mm), along with the geographical centroids of each county in Iowa (latitude and longitude). Feature engineering is defined as selecting, manipulating, and transforming primary data into features utilized in supervised machine learning (Zheng and Casari, 2018) and was used in this study. Using the precipitation, temperature, and location data, fungal growth data were calculated using equations from Battilani et al. (2013), as shown in Eqs 1, 2. These equations have been applied to Illinois, a neighboring state of Iowa in the US with similar environmental conditions (Castano-Duque et al., 2022).


A=5.98

B=1.70

C=1.43

$$T_{\max}=48$$

$$T_{\min}=5$$

(1) $$T_{\mathrm{eq}}=\frac{\left(T_{\mathrm{average}}-T_{\min}\right)}{(T_{\max}-T_{\min)}}$$

(2) $$\mathrm{Growth}={\left[A\times \left(T_{\mathrm{eq}}^{B}\right)\times \left(1-T_{\mathrm{eq}}\right)\right]}^{C}$$

*T*_eq_ is calculated per month (Eq. 1). A weighted fungal growth (10% of the original growth) was used for months without corn in the field, including January–April and November–December, as it was an assumption in the model. The AFL production index was calculated using Eqs 3, 4 from Battilani et al. (2013) research.


A=4.84

B=1.32

C=5.59

$$T_{\max}=47$$

$$T_{\min}=10$$

(3) $$T_{\mathrm{eq}}=\frac{\left(T_{\mathrm{average}}-T_{\min}\right)}{(T_{\max}-T_{\min)}}$$

(4) $$\mathrm{AFL}={\left[A\times \left(T_{\mathrm{eq}}^{B}\right)\times \left(1-T_{\mathrm{eq}}\right)\right]}^{C}$$

*T*_eq_ was calculated per month (Eq. 3). The model utilized an ON/OFF switch for dispersal (Battilani et al., 2016; Castano-Duque et al., 2022), and it assumed dispersal was ON if there was less than 127 mm of accumulated rain per month. If more than 127 mm of accumulated rain per month, dispersal was assumed OFF (Castano-Duque et al., 2022). A featured engineered equation was produced to calculate the ARI in fields during the month corn was present (Eq. 5).


(5) ARI=growth×dispersal×AFL

During the months where no corn was present in the fields (January–April and November–December), ARI was calculated as presented in Eq. 6.


(6) $$\mathrm{ARI}={\mathrm{weighted}}_{\mathrm{growth}}\times \mathrm{dispersal}$$

The weighted growth was an assumption in the model that takes only 10% of the fungal growth when no corn is in the field. Model predictors were monthly ARI through the noted years in each county in Iowa.

The input features of the monthly vegetative index per county were generated by satellite data acquired from GRO-Intelligence Company, and this was the secondary featured engineered variable. For the missing values in monthly ARI and vegetative index predictors, imputation was performed using predictive means models (pmm) (*mice*; the mean method was used; Heitjan and Little, 1991; Schenker and Taylor, 1996) in an R package (R Development Core Team, 2014). Imputation was able to determine plausible values from the distribution of missing data points. The mice algorithm fills in a value randomly among the observed donor values from an observation whose regression-predicted values are closest to the regression-predictive value for the missing value from the simulated model (Heitjan and Little, 1991; Schenker and Taylor, 1996). Similar to Castano-Duque et al. (2022) study, the ARI was removed from January, February, and December. The AFL data was linked to the feature data set to create 630 observations and 70 predictors, excluding the independent validation year 2020 (*n* = 376).

### 2.3. Output variables and correlation analysis

The output values for AFL were categorized based both on FDA’s action levels for corn in general commerce or unknown end use (20-ppb) and lower thresholds based on global standards (5-ppb) (FDA, 2000; EFSA, 2013). For AFL, a high category was considered for contamination levels greater than 20-ppb and low for levels 20-ppb or less (Supplementary Table S2). A secondary analysis was incorporated to reduce the risk threshold for high contamination levels greater than 5-ppb and low levels of 5-ppb or less to determine similarities or differences in output variables (Supplementary Table S2). A correlation analysis was performed among all the predictors and output variables using a confidence level of 0.95 for correlation and hclust method based on Pearson and Spearman correlation (*corfunction*; R Development Core Team, 2014).

### 2.4. Gradient boost machine learning for AFL

ARI for January, February, and December were excluded from the model as these months had too many missing values to be imputed. The GBM software package in R provided extensions to Freund and Schapire’s AdaBoost algorithm and Friedman’s gradient boosting machine (GBM) learning (Friedman, 2001). For performing GBM, Iowa’s county identifier was removed from the data set, then partitioned for training and testing using a 90%–10% ratio.

The predictors used for AFL-GBM were the monthly ARI, weather data, vegetation index, and soil properties. The following flags on the training data were used for AFL: a threshold of 500 trees, interaction depth of one, shrinkage of 0.01, 10 cross-validation folds, and the distribution was selected as multinomial (Supplementary Table S3). The GBM package performed prediction analysis using the testing data set and the best fit generated from the training data. A confusion matrix was developed using the caret package in R that computed the overall statistics. The GBM package computed the effect values for each predictor in the model.

### 2.5. Validation using 2020 AFL data and GBM

The GBM software package in R was used to perform prediction analysis using the 2020 AFL data set, and the best number of trees was determined by the training data set (Friedman, 2001). Validation was done using the best fit of GBM for AFL and generated from the training data. The weather and AFL data for 2020 were prepared as described in the methods section. The AFL data for 2020 included 99 counties and 376 observations.

## 3. Results

### 3.1. AFL contamination in Iowa

This study obtained 630 observations of AFL contamination levels (high and low) in Iowa corn from historical surveys conducted by the IDALS in 2010, 2011, 2012, and 2021 (excluding 2020 for independent validation) (Table 1). From the overall historical dataset, AFL contamination in corn had 2.30% of samples with high contamination levels (>20 ppb) and 97.70% with low levels (≤20 ppb) for the first risk threshold. The second risk threshold had AFL contamination in corn with high contamination levels (>5 ppb) at 7.10 and 92.90% with low levels (≤5 ppb). AFL contamination levels were highest in 2012 when there was a known historic drought event in Iowa (Mitchell et al., 2016; Table 1). Otherwise, AFL contamination in Iowa inflated with ≤5 and ≤ 20 ppb was considered a rare event, making it difficult for the model to detect high contamination levels due to the low incidence rate, thus, decreasing the model’s accuracy.

**Table 1:** Distribution of AFL contamination in Iowa over 5 years for both risk thresholds (including 2020, which was used for internal validation).

Summary statistics of AFL observations in Iowa				5-ppb risk threshold			20-ppb risk threshold		
Year	*n*	Mean	SD	High (#)	Low (#)	Prevalence of high threshold (%)	High (#)	Low (#)	Prevalence of high threshold (%)
2010	49	0.09	0.60	0	49	0.00	0	49	0.00
2011	89	0.48	1.88	1	88	1.14	0	89	0.00
2012	388	5.04	16.97	67	321	20.87	22	366	6.01
2020	376	0.40	6.54	3	373	0.80	1	375	0.27
2021	104	0.01	0.09	0	104	0.00	0	104	0.00

### 3.2. Weather predictors and feature engineering

**Figure 1:**
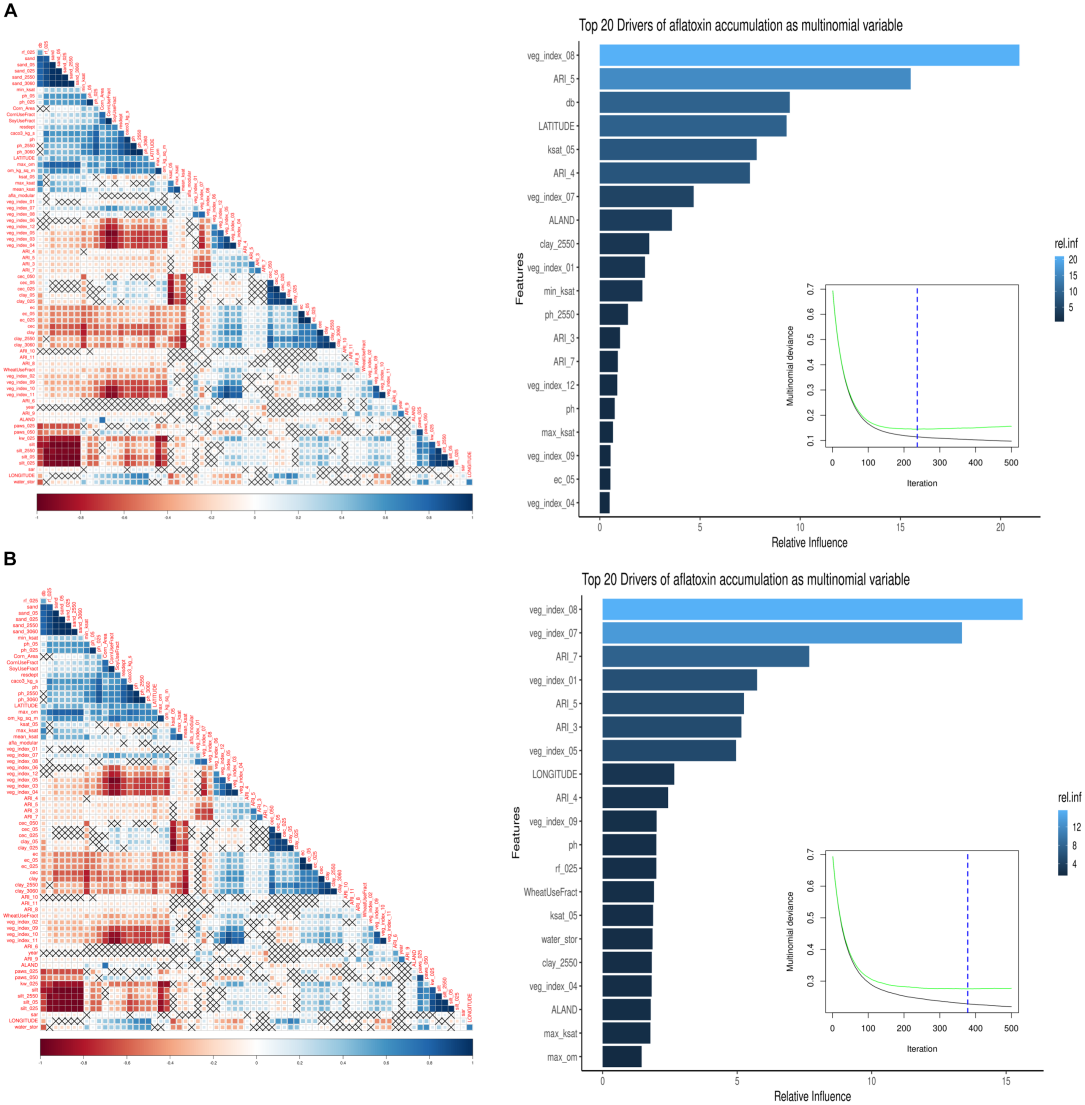
Summary of the GBM model using multinomial AFL outcomes. The left image is a pair-wise correlation analysis of all the model predictors for AFL using the hclust method. The correlation level is depicted from positive correlation (blue) to negative correlation (red); black crosses represent non-significant *p*-values of correlation analysis between all predictors. The *p*-values cut-off was 0.05, and the confidence level was 0.95. The right image summarizes the GBM model using multinomial AFL outcomes, showing the number of iterations where cross-validation error is minimized, and the relative influencing parameters of AFL contamination in Iowa corn. The AFL model used an interaction depth of 1, shrinkage of 0.01, and 10 c.v. folds. The top 20 influential predictors and their relative influence within the model for predicting AFL. The blue hue represents levels of the relative influence of the predictors, where light blue has high and dark blue has low influences. **(A)** Pair-wise correlation for 20-ppb threshold, 237 total iterations, and 26 predictors being a non-zero influence. **(B)** Pair-wise correlation for 5-ppb threshold, 378 total iterations, and 53 predictors being a non-zero influence.

The monthly ARI for AFL was the primary feature-engineered predictor created by employing multiple mathematical functions that linked plant-fungal interactions with biological relationships, weather parameters, and crop developmental markers (Battilani et al., 2013; Van der Fels-Klerx et al., 2019; Liu et al., 2021). Feature engineering decreased predictor variables and correlation levels among meteorological predictors in the model; therefore, decreased overfitting reduced the high correlation among the predictors (Castano-Duque et al., 2022; Figure 1). Ensemble methods is a machine learning technique combining several base models to produce one optimal predictive model (Dietterich, 2000). The low overfitting of the model was done using GBM; the model ensembles data and can learn from previous errors during the ensemble (Cooper, 1990; Friedman, 2001). The vegetative index was obtained from satellite imaging, allowing the model to include plant greenness of the vegetation on the earth’s surface (Xue and Su, 2017; Castano-Duque et al., 2022).

### 3.3. GBM analysis for AFL

GBM prioritized predictors that allowed the model to be run during the corn growing season, including pre-planting, planting, plant growth and development, flowering, and harvest (Castano-Duque et al., 2022). GBM was used to model AFL contamination levels in corn, as Castano-Duque’s et al. (2022) model had the highest accuracy for GBM versus Bayesian networks. ARI predictors after harvest (November) were removed from the predictors, as corn was absent in the field. The model could predict both contamination levels (high and low; Table 2). The optimal number of trees for the model of the 20-ppb threshold was 237 and 378 for the model of the 5-ppb threshold, representing the number of trees where cross-validation error is minimized (Figure 1). The McNemar *value of p* for the GBM model was 0.4795 for the 20-ppb threshold and 0.6831 for the 5-ppb threshold (Supplementary Table S3). The overall specificity for high AFL contamination levels in corn for the 20-ppb threshold was 1, where the sensitivity was 0. Compared to the 5-ppb threshold, the overall specificity for high AFL contamination levels in corn was 0.96, where the sensitivity was 0.33. The GBM had an acceptable specificity; however, the overall sensitivity was low. This could be due to the differences in the proportionality of high and low contamination levels in the prediction of output variables. The overall accuracy of the 20-ppb GBM-AFL model was 96.77%, whereas the balanced accuracy for both high and low contamination levels was 50.00% (Table 3). The overall accuracy of the 5-ppb GBM-AFL model was 90.32%, whereas the balanced accuracy for both high and low contamination levels was 64.88% (Table 3). The GBM model had a low power to detect high-level AFL contamination events for both a 20- and 5-ppb risk threshold. The multi-class area under the curve was 0.50 for 20-ppb and 0.65 for 5-ppb, respectively (Figures 1A,B). This was used to evaluate the classifier and distinguish between high and low contamination events.

**Table 2:** Confusion matrix of multinomial outcomes for AFL-GBM analysis of both thresholds to validate reference testing data (10%) after training with actual data for toxin levels and predicted levels using the model (90%).

AFL (*n* = 62)		High > 20 ppb	Low ≤ 20 ppb	Total predicted	High > 5 ppb	Low ≤ 5 ppb	Total predicted
		High	Low		High	Low	
Prediction	High	0 (0.00%)	0 (0.00%)	0 (0.00%)	2 (3.22%)	2 (3.22%)	4 (6.45%)
	Low	2 (3.22%)	60 (96.77%)	62 (100%)	4 (6.45%)	54 (87.10%)	58 (93.55%)
Total actual		2 (3.22%)	60 (96.77%)	62 (100%)	6 (9.68%)	56 (90.32%)	62 (100%)

**Table 3:** Accuracy statistics for GBM for AFL in Iowa-grown corn.

GBM Parameters for AFL		
	20-ppb threshold	5-ppb threshold
Accuracy	0.9677	0.9032
95% Confidence interval	(0.89, 0.97)	(0.80, 0.96)

**Figure 2:**
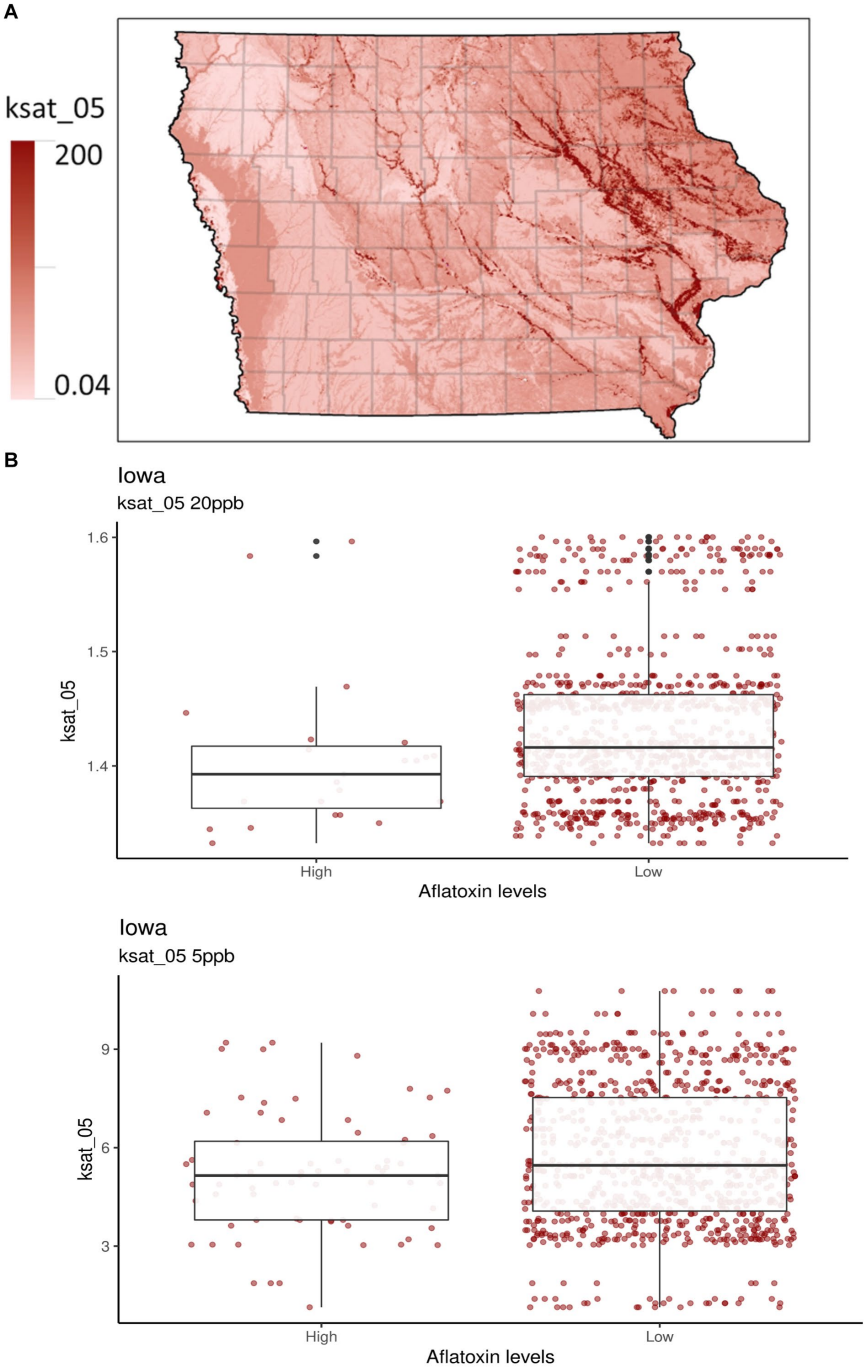
Selected soil properties of Iowa. Ksat_05 measures the saturated hydraulic conductivity from the soil surface to 5 cm depth in units of μm sec^−1^. **(A)** The gradient graph is the Ksat_05 across Iowa. **(B)** The boxplots are for Ksat_05 with high and low AFL contamination levels for 20- and 5-ppb risk thresholds, respectively.

For the 20-ppb AFL risk threshold, 26 of the 70 predictors for the GBM model had a non-zero influence. Among the 26 predictors, the top five were: (1) Vegetative index in August, (2) ARI in May, (3) Bulk density in soil (g/cm^3^), (4) Latitude, and (5) Saturated hydraulic conductivity (Ksat) (Figures 1A, 2). For the 5-ppb AFL risk threshold, 53 of the 70 predictors for the GBM model had a non-zero influence. Among the 53 predictors, the top five were: (1) Vegetative index in August, (2) Vegetative Index on July, (3) ARI in July, (4) Vegetative Index in January, and (5) ARI in May (Figure 1B). Vegetative index relates to the greenness degree of all plants and soil captured by satellite imaging; our results showed that vegetative index in August is the most significant feature in the model to predict AFL contamination. An inverse relationship exists between vegetation index in August and AFL contamination (Figure 1); thus, a higher index, greener “healthy” plants, leads to lower AFL. August is environmentally and ecologically significant because if there are drought concerns in Iowa during August, there would be reduced values of the vegetative index (low greenness levels), signaling increased AFL contamination at harvest. The summary statistics can be found in Table 3.

### 3.4. Model validation

The 20-ppb and 5-ppb models were validated to understand the model’s predictive capacity using the 2020 AFL data that included 99 counties and 376 observations. High levels of AFL contamination were rare among the 376 observations from 2020, with only 1 observation above the 20-ppb threshold (0.27%) and 3 observations above the 5-ppb threshold (0.80%). GBM successfully predicted low AFL contamination levels with 99.73% accuracy for the 20-ppb risk threshold, whereas a 5-ppb risk threshold predicted low AFL contamination at 99.20%. The models could not predict a single observation of high AFL contamination for 2020 (Table 4).

**Table 4:** Confusion matrix of multinomial outcomes for AFL-GBM analysis for both thresholds to validate reference testing data set to actual data for toxin levels and predicted levels using the 2020 validation set.

AFL (*n* = 376)		High > 20 ppb	Low ≤ 20 ppb	Total predicted	High > 5 ppb	Low ≤ 5 ppb	Total predicted
		High	Low		High	Low	
Prediction	High	0 (0.00%)	0 (0.00%)	0 (0.00%)	0 (0.00%)	0 (0.00%)	0 (0.00%)
	Low	1 (0.27%)	375 (99.73%)	376 (100%)	3 (0.80%)	373 (99.20%)	376 (100%)
Total actual		1 (0.27%)	375 (99.73%)	376 (100%)	3 (0.80%)	373 (90.20%)	376 (100%)

## 4. Discussion

AFL is of great concern to the US corn industry, as it poses a significant food and feed safety risk to humans and livestock due to the detrimental effects of being a known class 1a carcinogen (Eaton and Gallagher, 1994; CAST, 2003; Mitchell et al., 2016; Yu et al., 2022). Additionally, AFL has significant cost implications for agricultural and food economies in the US and even globally due to the far-reaching contribution of US corn (Mitchell et al., 2016). In this case study, an Iowa-centric model was developed to predict AFL contamination using historical corn contamination, meteorological data, and soil property data in the state producing the largest amount of corn in the US (USDA-NASS, 2023a,b). This research follows Castano-Duque’s model that predicted AFL and FUM contamination in Illinois corn, a neighboring state to Iowa (Castano-Duque et al., 2022). The GBM model was selected for analysis due to the nature of the data, with GBM performing better for AFL contamination in the Illinois-centric model (Castano-Duque et al., 2022). The AFL risk values were set using US FDA regulations in FSMA, where corn entering general commerce has an action level of 20-ppb (FDA, 2000). The risk level was reduced to 5-ppb in this study to compare with global standards that pose more stringent AFL regulations (Wu and Guclu, 2012; EFSA, 2013; Wu, 2015). The AFL-GBM had an overall accuracy of 96.77% for the 20-ppb risk threshold and 90.32% for the 5-ppb risk threshold. Using GBM, predictors that significantly influenced the model could be determined. The predictor analysis indicated several meteorological events and soil properties before planting and during corn growth that strongly influenced predictions of AFL during harvest. The ability to predict AFL contamination while corn is in the field signifies preventative versus reactive management of mycotoxin outbreaks, following FSMA’s overall goal for food and feed safety (Grover et al., 2016; King and Bedale, 2017).

The AFL-GBM model with a threshold of 20-ppb had adequate overall accuracy; however, the balanced accuracy was 50.00% for high and low contamination events. With only 4 years of historical Iowa AFL contamination data, only 2.30% of high contamination levels were above the regulatory limits of 20-ppb in the full historical database. When the risk threshold values were reduced to 5-ppb for AFL’s high and low contamination levels, the balanced accuracy was increased to 64.88%. The increased balanced accuracy was due to the enhanced amount of AFL contamination events in Iowa at 7.10%, therefore, including more observations of high AFL contamination due to the lower threshold. Compared to the published Illinois-centric AFL-GBM, which had a balanced accuracy of 61% for high, 54% for medium, and 60% for low contamination levels (Castano-Duque et al., 2022), the Iowa-centric AFL-GBM balanced accuracy for 20-ppb threshold was reduced (50%, Table 3) due to a lower incidence of AFL contamination events and a reduced overall total amount of observations available for the training data set. The reduced sensitivity with the GBM model for both thresholds indicates it has a low power to predict high AFL contamination events. Future research is needed to fine-tune the model to enhance the sensitivity. Cheng et al. (2019) suggest enriching the sample set with higher AFL contamination observations or adjusting the algorithm to penalize high false negative rates for improving the overall balanced accuracy of predictive models (Krawczyk, 2016). These results differ significantly from European models that show >75% general accuracy for corn in multiple regions (Battilani et al., 2013; Leggieri et al., 2021). If the risk value were reduced, as shown above, the model would have a higher specificity because the model would have the ability to learn from more balanced data (Cooper, 1990; Friedman, 2001; Natekin and Knoll, 2013).

The Iowa-centric GBM model included 70 predictors, with 26 having non-zero influence for AFL at 20-ppb and 53 for 5-ppb, respectively. The highest influence for AFL-GBM was the vegetative index in August for both risk thresholds (Supplementary Table S2). Like Castano-Duque et al. (2022)’s Illinois-centric model, weather data was acquired to help perform feature engineering with mechanistic mathematical equations to determine AFL production from *Aspergillus* growth. The vegetative index, a satellite-acquired data type, also known as the NDVI, helps distinguish visible red and near-infrared reflectance bands, allowing for the identification of vegetation, soil, water, and other features (Gro-Intelligence, 2019). NDVI reports plant greenness by indirectly measuring chlorophyll content and photosynthetic activity and behaving as a proxy of plant health, biomass, and yield (Wang et al., 2016; Gro-Intelligence, 2019). The vegetative index in August had the highest relative influence in the model for both risk thresholds; therefore, the results agreed that plant greenness in August was a significant determinant of AFL contamination at the time of harvest (Castano-Duque et al., 2022; Supplementary Figure S1). In August, on average, corn in Iowa should be approximately 8 feet tall with reasonably high vegetative indices due to sufficient plant greenness (Gro-Intelligence, 2019; USDA-NASS, 2023a,b). Suppose the NDVI index is low; this could be a diagnostic for drought and other crop stressors that might not be visible; this event could potentially predict AFL contamination due to fungal outbreaks during pre-harvest (Kerry et al., 2017; Gro-Intelligence, 2019). The vegetative index in July was also in the top five influential factors for AFL-GBM for the Iowa-centric model with the 20-ppb threshold and the seventh for the 5-ppb threshold, which is comparable to the Illinois-centric model (Castano-Duque et al., 2022). Therefore, NDVI may enhance AFL prediction preharvest in the late summer months in the Midwest Corn Belt due to the potential presence of detecting corn plant stress (Wang et al., 2016; Kerry et al., 2017).

Another top influential factor in the model was ARI in May, the second most influential feature for determining AFL contamination at the end of the growing season for the 20-ppb risk threshold (Supplementary Figure S2). During May, on average, in Iowa, corn is in the vegetative growth stage (USDA-NASS, 2023a,b). Higher ARI in this month was linked to the prediction of high AFL contamination levels; thus, it agrees with Yu et al. (2022) that warmer weather early in the planting and growing season leads to higher AFL contamination levels during harvest (Castano-Duque et al., 2022). For the 5-ppb risk threshold, ARI in July and May were the top influential factors. These findings agreed with the Illinois-centric model for early months having a high relative influence on predicting AFL contamination; therefore, agronomic practices that happen when corn is not in the field, such as tilling and drilling, should be further researched to determine if fungal growth for AFL is being harbored in soil residues (Accinelli et al., 2008; Abbas et al., 2009; Herrera et al., 2023). Tillage practices are an essential pre-planting factor for determining *Aspergillus* spores in leftover crop stover; the chances for infection are greater if leftover stover is left on the soil (Payne et al., 1986; Herrera et al., 2023). Even though it is wise from a conservation standpoint to conduct no-till practices to maintain soil resources, it may enhance AFL contamination (Abbas et al., 2009). Furthermore, alternative cover crop management may be allelopathic to *A. flavus* and *parasiticus* and should be further researched to create pest management practices that could be conducive to lower AFL contamination risk while maintaining no-till practices (Abbas et al., 2009; Damianidis et al., 2018).

Additionally, latitude as a predictor in the 20-ppb risk threshold AFL-GBM showed a high influence on contamination levels at the end of the year (Supplementary Table S2). Although high AFL contamination in Iowa was rare (2.30%), these events primarily occurred in the southern portion of the state at lower latitudes. This has been documented in previous studies, where the areas of Iowa that had the highest contamination values nearing the 20-ppb threshold were the Southwest and South-Central Crop Reporting Districts in Iowa (Mitchell et al., 2016). Similar results for AFL contamination at lower geographic latitudes were found in 1983 and 1989 in Iowa (Schmitt and Hurburgh, 1989; Russell et al., 1991). A unique finding in this study is that when the risk index values for AFL were reduced from 20-ppb to 5-ppb for high and low contamination events, latitude was removed from the top 20 influential predictors of the GBM model (Supplementary Table S2). It is hypothesized that the difference between latitude being in the top 20 influential factors for the model with the 20-ppb AFL regulatory limits is common cause versus special cause variation (MacGregor and Kourti, 1995). The 20-ppb model includes latitude as a factor for AFL contamination as a special cause variation; thus, the variation is unusual and unexpected, pointing to unique weather events such as drought or other crop stressors in the lower latitude in Iowa for higher AFL contamination values (MacGregor and Kourti, 1995). The 5-ppb model excludes latitude, as it is seen as a common cause variation, where it is expected and a consistent range of values with no pattern (MacGregor and Kourti, 1995). This finding agrees that low levels of AFL are commonly found in Iowa, even during years that are not conducive to producing AFL fungi (Zuber and Lillehoj, 1979; Munkvold et al., 2019).

A new addition to the Iowa-centric model was soil property predictors, which were indicated as a potentially influential factor for predicting AFL preharvest in the Illinois-centric model (Castano-Duque et al., 2022; Supplementary Table S1) and have been used in Europe-centric models (Leggieri et al., 2021). The top two influences for the 20-ppb threshold for soil properties were bulk density (db) (g/cm^3^) and saturated hydraulic conductivity (Ksat), 5 cm depth (μm/s^−1^). For the 5-ppb threshold, the top two influences for soil properties were pH and rock fragments (% by volume from 0 to 50 cm) (Supplementary Table S2). Ksat was third in relative influence compared to the 20-ppb risk threshold (Figure 2). Ksat measures the water flow rate through saturated soil at a given hydraulic gradient. It relates to the water-holding capacity, the level of soil compaction, soil db, and texture and influences the propensity for soils to become water-logged in moist conditions (Vauclin et al., 1994; Brady and Weil, 2002; Libohova et al., 2018). Ksat and db are directly correlated, as both predictors affect water movement in the soil (Laboski et al., 1998; Rahimi et al., 2011; Figure 1). High db indicates low soil porosity and compaction, which may restrict root growth, air, and water movement (Blake, 1965; Supplementary Figure S3). Furthermore, soil pH is considered a crucial soil predictor as it broadly influences many soil processes, including nutrient and micro-nutrient availability, species richness of fungi, plant growth, acidification processes, cation exchange capacity, redox potential, and plant diseases (Thomas, 1996; Winter and Pereg, 2019; Baltensweiler et al., 2020). *A. flavus* and *parasiticus* have an optimum growth pH between 3.5 and 8 (Wheeler et al., 1991; Winter and Pereg, 2019). Soil pH is correlated with db, indicating an increase in pH with increased db (Li et al., 2020). To our knowledge, no studies have been published regarding the relationship between Ksat, db, pH, and *A. flavus* or *parasiticus* fungal growth for AFL production. Therefore, our results indicate that soil types and textures may be one of the main drivers in determining whether AFL in Iowa will be a high or low contamination year (Brady and Weil, 2002; Kerry et al., 2022).

These findings for the influential factors for both risk thresholds showcase that AFL contamination consists of a multi-traffic network, requiring interactions among the fungi, corn plant, and environmental conditions consisting of climate, weather, and soil properties (Klich, 2007; Abbas et al., 2009). This case study aimed to determine a baseline for interactions of these influential factors at two AFL risk thresholds of 20- and 5-ppb, respectively. Providing an Iowa-centric model that can predict AFL risk values pre-harvest to stakeholders in the context of the largest corn production region in the US enables early actions to prevent or hinder at-risk corn for individuals conducting hazard analyses and risk assessments. Stakeholders can take preventative or mitigative procedures that are controllable such as isolating AFL-contaminated grain, increasing fungicide application, early harvest, increased drying for lower storage moistures, and strategic marketing to end-users with higher tolerances such as beef cattle (Widstrom et al., 2003; Fumagalli et al., 2021). For grain elevators, handlers, and processors, the Iowa-centric model can be used in a proactive mycotoxin monitoring program, paving the way for strategic and targeted sampling and testing of corn (Park and Troxell, 2002; Whitaker, 2003).

Further research is warranted to understand what may cause corn plants to become susceptible versus non-susceptible to AFL infection based on the top influential factors provided by the GBM model. From a practical and management perspective, AFL contamination in corn can only be controlled to a certain degree. Stakeholders cannot fix uncontrollable factors for AFL contamination, such as certain climate or weather parameters; however, agronomic or management practices can mitigate AFL contamination for controllable influences, such as the management practices listed above (Fumagalli et al., 2021).

In conclusion, this study demonstrated that developing a predictive model for AFL in Iowa corn with historical contamination data, meteorological data, and soil properties had high accuracy at 96.77% for 20-ppb and 90.32% for 5-ppb, respectively. Comparable to the Illinois-centric model, the GBM model performed well by predicting low levels of AFL contamination; however, the balanced accuracy for high contamination values of AFL was reduced due to the rarity of high contamination events in Iowa. The vegetative index in August significantly influenced AFL risk for both thresholds, indicating that August is environmentally and ecologically important due to drought concerns. Additionally, soil property predictors, such as Ksat, pH, and db, may influence AFL contamination levels preharvest. Future work will finetune the Iowa model from county to macro-scale Crop Reporting District to enhance the data included to improve balanced accuracies and detect high AFL contamination levels. Applications of how to utilize the Iowa-centric model by stakeholders will be developed in collaboration with USDA-ARS.

## Data availability statement

The datasets presented in this article are not readily available because they are confidential, as requested by the Iowa Department of Agriculture and Land Stewardship. Requests to access the datasets should be directed to GM, gamosher@iastate.edu.

## Author contributions

EB-S performed data collection, preparation, contribution to biological relationships with the model, and manuscript preparation. LC-D performed data analysis, imputation, feature engineering, modeling, contribution to biological relationships with the model, and manuscript preparation. PO and EW provided soil property parameters, biological relationships with the model, and manuscript preparation. KR, CH, GM, and EB performed initial planning and support for collaborative work. All authors contributed to the article and approved the submitted version.

## Funding

Portions of this project were funded by USDA/NIFA award 2022-690008-36645.

## Conflict of interest

The authors declare that the research was conducted in the absence of any commercial or financial relationships that could be construed as a potential conflict of interest.

## Publisher’s note

All claims expressed in this article are solely those of the authors and do not necessarily represent those of their affiliated organizations, or those of the publisher, the editors and the reviewers. Any product that may be evaluated in this article, or claim that may be made by its manufacturer, is not guaranteed or endorsed by the publisher.

## Footnotes

^1^ https://www.ncdc.noaa.gov/cdo-web/search

^2^ https://gro-intelligence.com/

## Abbreviations

AFL: Aflatoxin
US: United States
GBM: Gradient Boosting Machine
ARI: Aflatoxin Risk Index
Ksat: Saturated Hydraulic Conductivity
FSMA: Food Safety Modernization Act
db: Bulk Density
FDA: Food and Drug Administration
NOAA: National Oceanic and Atmospheric Administration
NDVI: Normalized Difference Vegetative Index
