# Supplementary figures and images for: Gradient boosting machine learning model to predict aflatoxins in Iowa corn
Source: Front Microbiol. 2023 Sep 1;14:1248772. doi: 10.3389/fmicb.2023.1248772 (PMC10502509; doi:10.3389/fmicb.2023.1248772)

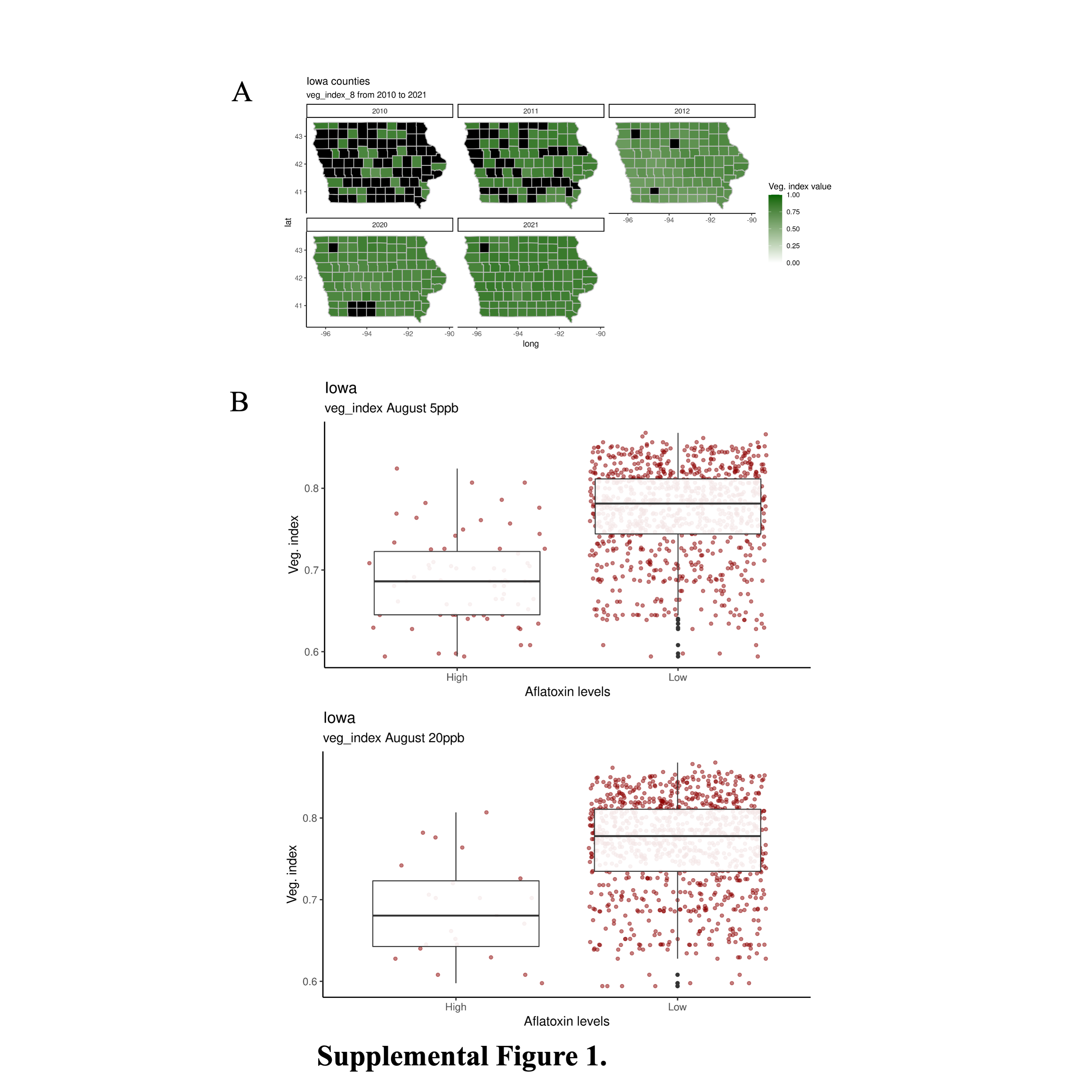

Supplement: Supplementary file 1 [file Image_1.TIFF]

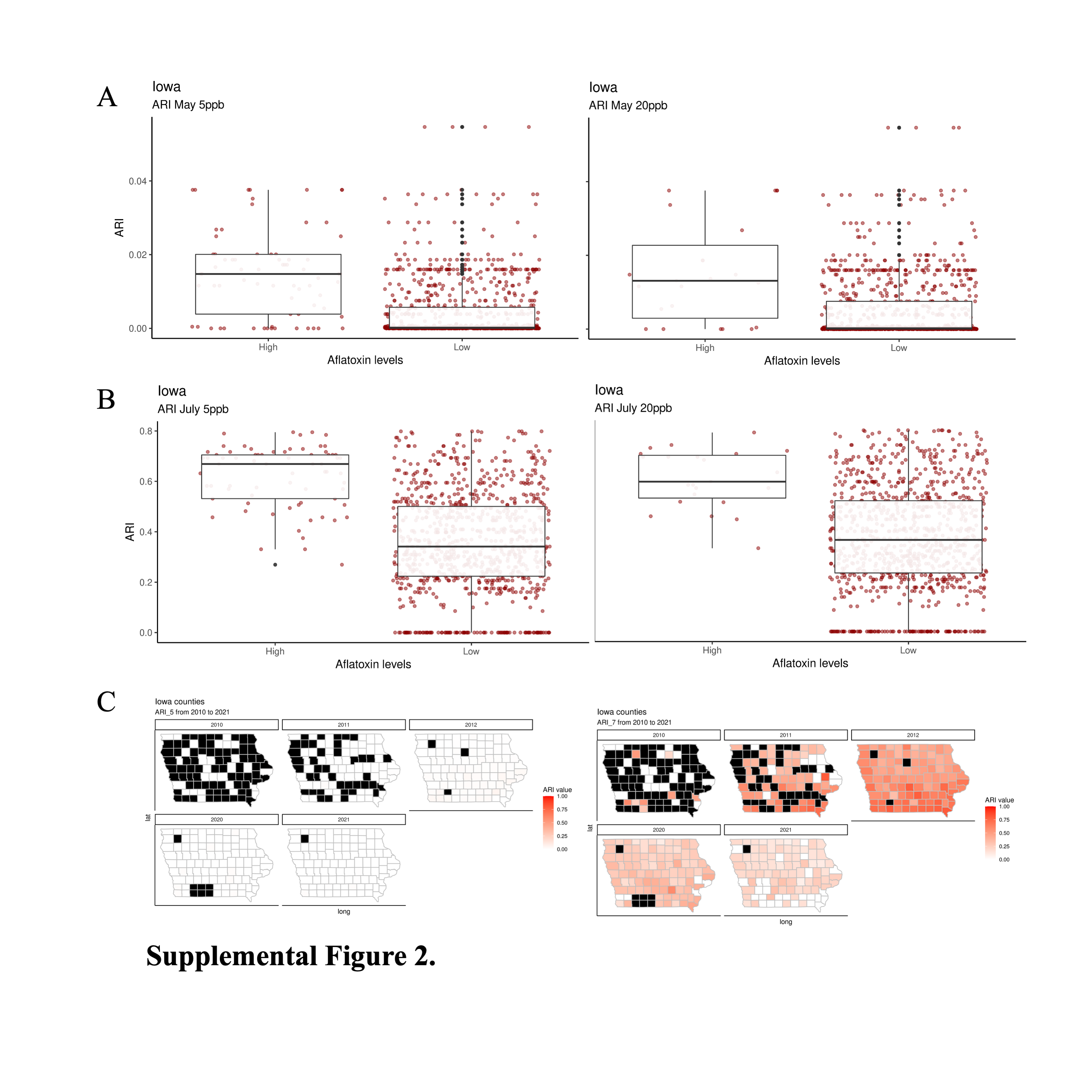

Supplement: Supplementary file 2 [file Image_2.TIFF]

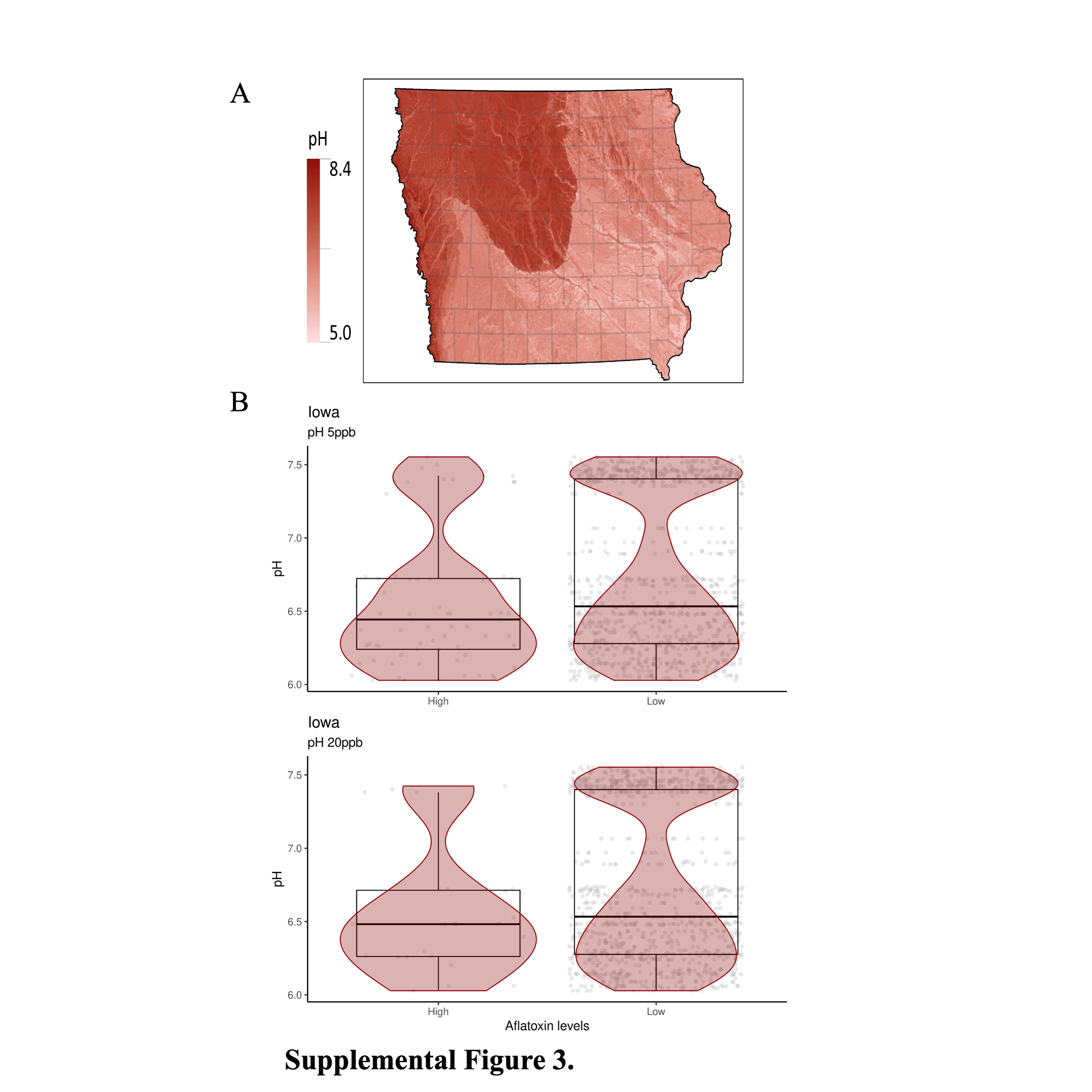

Supplement: Supplementary file 3 [file Image_3.TIFF]
